# Retraction: Silica nanoparticle acute toxicity on male *rattus norvegicus* domestica: ethological behavior, hematological disorders, biochemical analyses, hepato-renal function, and antioxidant-immune response

**DOI:** 10.3389/fbioe.2025.1727233

**Published:** 2025-11-28

**Authors:** Frontiers Editorial Office

The journal retracts the 4 April 2022 article cited above.

Following publication, concerns were raised regarding the integrity of the data in the published figure (2C), and the subsequent duplication of this same image - with contradictory labelling - in these four articles published between 2019 and 2023:

Karami E, Goodarzi Z, Shahtaheri SJ, Kiani M, Faridan M, Ghazi-Khansari M. The aqueous extract of Artemisia Absinthium L. stimulates HO-1/MT-1/Cyp450 signaling pathway via oxidative stress regulation induced by aluminium oxide nanoparticles (α and γ) animal model. BMC Complement Med Ther. 2023 September 5; 23(1):310. doi: 10.1186/s12906-023-04121-6.

Kiumarzi F, Morshedloo MR, Zahedi SM, Mumivand H, Behtash F, Hano C, Chen JT, Lorenzo JM. Selenium Nanoparticles (Se-NPs) Alleviates Salinity Damages and Improves Phytochemical Characteristics of Pineapple Mint (Mentha suaveolens Ehrh.). Plants (Basel). 2022 May 23; 11(10):1384. doi: 10.3390/plants11101384.

Zahedi SM, Moharrami F, Sarikhani S, Padervand M. Selenium and silica nanostructure-based recovery of strawberry plants subjected to drought stress. Sci Rep. 2020 October 19; 10(1):17672. doi: 10.1038/s41598-020-74273-9.

Shahrajabian, H., Sadeghian, F. The investigation of alumina nanoparticles’ effects on the mechanical and thermal properties of HDPE/rPET/MAPE blends. Int Nano Lett 9, 213–219 (2019). doi: 10.1007/s40089-019-0273-7

The authors failed to provide the raw data or a satisfactory explanation during the investigation, which was conducted in accordance with Frontiers’ policies. As a result, the data and conclusions of the article have been deemed unreliable and the article has been retracted.

This retraction was approved by the Field Chief Editor of Frontiers in Bioengineering and Biotechnology and the Chief Executive Editor of Frontiers. The authors have not responded to correspondence regarding this retraction.

Frontiers would like to thank Sholto David for contacting the journal regarding the published article.

